# Nonunion in Patients with Tibial Shaft Fractures: Is Early Physical Status Associated with Fracture Healing?

**DOI:** 10.7759/cureus.7649

**Published:** 2020-04-12

**Authors:** Raman Mundi, Daniel Axelrod, Diane Heels-Ansdell, Harman Chaudhry, Olufemi R Ayeni, Brad Petrisor, Jason W Busse, Lehana Thabane, Mohit Bhandari

**Affiliations:** 1 Surgery, McMaster University, Hamilton, CAN; 2 Orthopaedic Surgery, McMaster University, Hamilton, CAN; 3 Health Research Methodology, Biostatistics, McMaster University, Hamilton, CAN; 4 Orthopaedic Surgery, University of Toronto, Toronto, CAN; 5 Orthopaedics, Hamilton Health Sciences, Hamilton, CAN; 6 Health Research Methodology, McMaster University, Hamilton, CAN

**Keywords:** non union, trauma, fracture healing, tibia fractures

## Abstract

Background

Nonunions of tibial shaft fractures have devastating physical and psychological consequences for patients. It remains unknown if early functional status can identify patients at risk for nonunion.

Questions/Purposes

To determine if functional status at three months after surgery, as measured by either the short form 36 (SF-36) or the short form 12 (SF-12) health survey physical component summary (SF-12 PCS) score, can serve as a prognostic indicator for nonunion at one year in patients with fractures of the tibial shaft.

Patients/Methods

This study was an observational cohort study nested within two multicenter, randomized controlled trials. Patients who met the following eligibility criteria were included: (1) sustained a tibial shaft fracture that was treated with intramedullary nailing, (2) were unhealed at the three-month follow-up, (3) had a reported SF-36 or SF-12 PCS score at three months, (4) had the final 12-month follow-up with a reported radiographic healing status (bone union or nonunion), and (5) were enrolled in either the Study to Prospectively Evaluate Reamed Intramedullary Nails in Patients with Tibial Shaft Fractures (SPRINT) or Fluid Lavage of Open Wounds (FLOW) randomized trials. Multivariable logistic regression was performed to evaluate the association between healing status at 12 months and seven prognostic variables (open fracture, fracture pattern, nailing technique, smoking, fracture gap, three-month PCS score, and FLOW vs. SPRINT trial).

Results

A total of 940 patients were included in this study with an overall rate of radiographic nonunion of 13.3% (n=125) at the 12-month follow-up. Absolute nonunion risk increased with incrementally lower PCS scores (8.2%, 12.8%, 15.9%, 23.7% for scores ≥ 40, 30.0-39.99, 20.0-29.99, and < 20, respectively). In the multivariable regression analysis, PCS scores of < 20 were associated with a 2.6-times greater odds and 10% absolute risk increase of non-union, as compared to scores of ≥ 40 (OR 2.58, 95%CI: 1.02-6.53, ARI: 10.3, 95% CI: 0.1 - 28.2), whereas scores between 20 and 30 were associated with a nearly two-times greater odds of nonunion and a 6.4% absolute risk increase of nonunion (OR 1.94, 95%CI: 1.08-3.49, ARI: 6.4, 95% CI 0.6 - 15.3). Open fractures also conferred a 2.8-fold increase in odds of nonunion as compared to closed injuries (OR 2.77, 95%CI: 1.58-4.83), as did complex fractures when compared to simple fractures (OR 2.57, 95%CI: 1.64-4.02).

Conclusion

A considerable portion of patients with fractures of the tibial shaft treated with intramedullary nailing will experience nonunion at one-year postoperatively. Nonunion can be accurately predicted by patient functional recovery at three months as measured by the PCS of the SF-36 and SF-12 instruments.

## Introduction

Tibial shaft fractures represent the most common fractures amongst long bones, with an annual incidence of approximately 20 per 100,000 people in the developed world [1-3]. Despite modern surgical techniques of intramedullary nailing for the fixation of these fractures, a considerable number of patients fail to heal (15%-19%) and experience significant physical hardship and psychological suffering as a result of nonunion [4-6]. Nonunions of tibial shaft fractures also impose a financial burden on health care systems, as the management of such patients is associated with a greater than two-fold increase in health care costs as compared to patients without nonunion [7].

The capacity to identify patients at risk of nonunion early in their healing course would be of substantial value to orthopedic surgeons in initiating appropriate surveillance and possible intervention for such patients. To date, most variables that have been delineated as prognostic factors that influence healing have been baseline characteristics such as smoking, skin integrity, degree of cortical continuity, and intramedullary nailing technique [4-5,8-12]. It is likely, however, that a patient’s early healing response may be a more potent predictor of healing potential than such baseline characteristics. For example, Lack et al. reported that a radiographic assessment demonstrating any cortical bridging within four months postoperatively is predictive of eventual fracture healing with 99% accuracy [13].

Given the significant physical impairment associated with fracture nonunion, early functional recovery may potentially serve as a strong marker for healing potential [6,8]. The short form 36-Item (SF-36) health survey, along with its shortened version, the short form 12-item (SF-12) health survey, are generic health-related quality of life instruments that have found widespread use in the medical literature [14-15]. Both the SF-36 and SF-12 provide a general measure of a patient’s physical and mental health measured across eight assessment scales, including physical functioning, role limitations due to physical health, bodily pain, perceived general health, vitality, social functioning, role limitations due to emotional burden, and mental health. These scales can be aggregated to provide summary measures of overall physical and mental health, represented as the physical component summary (PCS) and mental component summary (MCS) scores, respectively. The PCS score is weighted more heavily on the first four above-mentioned health scales that relate to functioning [15-16]. As an inexpensive and time-efficient assessment tool that can be readily administered to patients, the SF-36 and SF-12 instruments could be of significant value in identifying patients at high risk for the failure of fracture healing.

To that end, we performed an observational study of patients with tibial shaft fractures treated with intramedullary nailing to determine if functional recovery at three months after surgery, as measured by the SF-36 and SF-12 physical component summary score, can serve as a prognostic indicator of nonunion at one year.

## Materials and methods

Study design

This study was an observational cohort study nested within two multicenter, randomized controlled trials, including the Study to Prospectively Evaluate Reamed Intramedullary Nails in Patients with Tibial Shaft Fractures (SPRINT) and the Fluid Lavage of Open Wounds (FLOW) trial [17-18].

SPRINT trial

The SPRINT trial was a randomized trial conducted across 29 centers in the United States, Canada, and The Netherlands, which compared reamed to unreamed intramedullary nailing in 1226 patients between July 2000 and September 2005. Enrolment criteria for the trial included skeletally mature patients with either open or closed fractures of the tibial shaft, which were nonpathological and amenable to intramedullary nailing. Patients in the SPRINT trial were prospectively followed for 12 months postoperatively, with functional outcomes assessed at three months using the SF-36 health survey and radiographic healing status assessed at 12 months.

The full SPRINT study protocol and study results have been previously published [17-18]. The trial received approval from the human subjects committee at each participating site (REB #99-077-Research Ethics Board/Institutional Review Boards).

FLOW trial

The FLOW trial was conducted across 41 sites from the United States, Canada, Australia, India, and Norway between June 2009 and October 2013. This randomized trial employed a 3x2 factorial design in which 2447 patients with open fractures were randomized to one of three irrigation pressures (high, low, very low) and to one of two irrigation solutions (soap vs. saline). Enrolment criteria for FLOW included skeletally mature patients with open fractures of any extremity requiring operative intervention. Among these patients, 929 had fractures of the tibial shaft. Patients were followed prospectively, with the SF-12 questionnaire administered at the three-month follow-up and radiographic healing status documented at follow-up visits for up to 12 months.

The FLOW study results and study protocol have been previously published, and the trial received approval from the human subjects committee at all participating centers (REB #08-268-Research Ethics Board/Institutional Review Boards) [19].

Inclusion criteria

All patients from the SPRINT and FLOW trials who met the following eligibility criteria were included in the current study: (1) sustained a tibial shaft fracture that was operatively treated with intramedullary nailing, (2) were unhealed at the three-month follow-up, (3) had a reported SF-36 or SF-12 PCS score at three months, and (3) had a reported radiographic healing status (bone union or nonunion) by the final 12-month follow-up.

Data collection and definitions of variables

For all patients, baseline data were retrieved and recorded for patient information (age, gender, ethnicity, smoking status, diabetic history, non-steroidal anti-inflammatory use), injury characteristics (mechanism, number of injuries, open versus closed fracture, fracture location, fracture pattern), and surgical factors (reamed versus unreamed nailing, postoperative fracture gap, time from injury to surgery).

In brief, the mechanism of injury was classified as either high- or low-energy, with high-energy injuries being inclusive of motor vehicle crashes (driver, passenger, or pedestrian), all-terrain vehicle (ATV)/snowmobile crashes, crush injuries, a fall from a height, and direct blunt trauma. Low-energy injuries included falls from standing, twists, and direct penetrating trauma. Open fracture wounds were graded using the Gustilo and Anderson classification. The fracture pattern was recorded as either simple (transverse, oblique, or spiral) or complex (comminuted or segmental). The fracture gap referred to the amount of bone loss between the proximal and distal fragments at the fracture site and was determined to be either < 1 cm or ≥ 1 cm from the postoperative radiographs.

The SF-36 and SF-12 PCS scores were recorded for patients in the SPRINT and FLOW trials, respectively. These surveys were either self-administered or interviewer-administered, if needed, at each patient’s three-month study follow-up visit in both trials. For both instruments, the PCS score ranges from 0 (worst possible function) to 100 (best possible function). PCS scores were categorized into the following strata based on scoring intervals of 10 or greater: <20.0, 20.0-29, 30.0-39, ≥40.0. Data were entered in these strata during the trial and thus could not be analyzed as a continuous variable in this secondary analysis. Categorization was performed to optimize the clinical relevance of our study findings by allowing for the reporting of absolute risks of nonunion per strata. It has been previously reported that the minimal clinically important difference (MCID) for the PCS score in an orthopedic population (osteoarthritis) is a score of two [20]. As such, the above intervals were deemed large enough to be clinically meaningful while allowing for a robust sample size of patients within each stratum based on frequency distributions.

The radiographic healing status of each patient in both trials was reported as either ‘yes’ (healed) or ‘no’ (unhealed) by the respective trial's adjudication committee, with an associated date of the first radiograph that showed healing. Radiographic interpretation of healing status was at the discretion of the clinical team at each specific site.

Data analysis

All baseline characteristics, functional scores, and radiographic outcomes are presented using descriptive statistics, consisting of means with associated standard deviations for continuous variables and frequencies with associated percentages for categorical variables. Multivariable logistic regression was performed to explore the association of the following seven factors with nonunion: three-month SF PCS scores, the trial to which the patient was enrolled (SPRINT or FLOW), and five covariates with previous evidence to suggest an association with fracture healing (skin integrity, fracture pattern, intramedullary nailing technique, smoking status, and fracture gap) [4-5,8-12,21]. Variables were entered into the regression model simultaneously.

It has been demonstrated that the SF-36 and SF-12 PCS scores are strongly correlated [22-23]. As such, patients from both trials were included in a single regression model. Nevertheless, a sensitivity analysis was performed in which an interaction term consisting of PCS score and study (FLOW vs. SPRINT) was added to the regression model to explore for an effect modification of the nonunion rate based on the instrument used (SF 36 vs. SF 12). An interaction term consisting of skin integrity (open vs. closed fracture) and IM nailing technique (reamed vs. unreamed) was also included in the sensitivity analysis, given prior evidence to suggest that the effect of the intramedullary nailing technique on the nonunion rate is dependent on skin integrity at the fracture site [10-11].

With a preliminary assessment of our study sample size (n=940) and an anticipated nonunion rate of approximately 15%, it was expected that all seven independent variables (six dichotomous and one four-level variable) could be included in the regression analysis without the risk of over-fitting the model [24]. All analyses were performed using IBM SPSS (Version 21; IBM Corp., Armonk NY). Statistical significance was set at a p-value of less than 0.05.

## Results

Results overview

**Figure 1:**
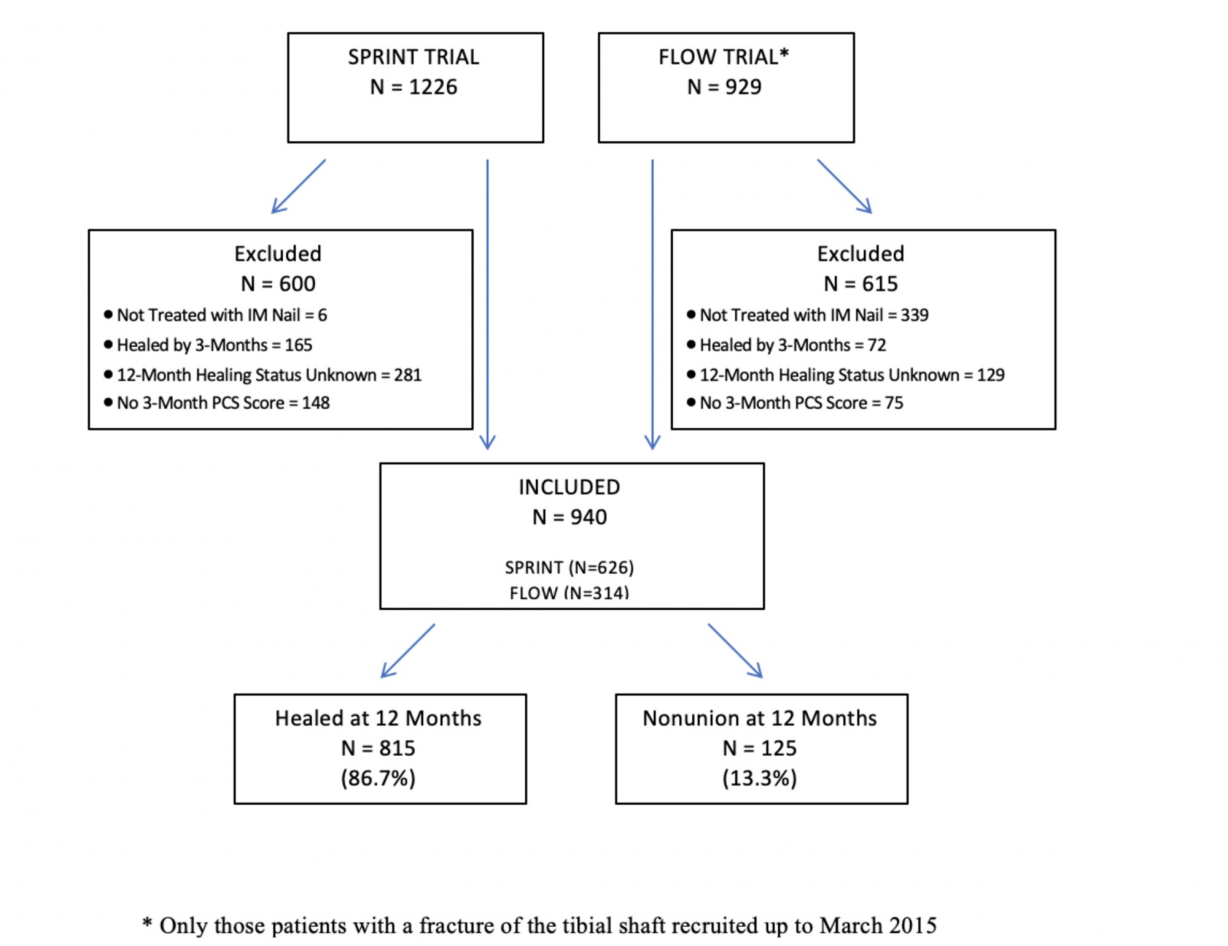
Study Flow Diagram

A total of 940 eligible patients with fractures of the tibial diaphysis were included in this study, with 626 patients incorporated from the SPRINT trial and 314 from the FLOW trial. The overall rate of radiographic nonunion at the 12-month follow-up was 13.3% (n=125). The rate of nonunion, when assessed independently for each trial cohort, was 10% for the SPRINT trial (64/626) and 19% (61/314) for the FLOW trial (Figure 1).

Patient, injury, and treatment characteristics

The study cohort comprised predominantly of young patients (mean age of 40.9) that were male (n=709, 75%) and of Caucasian descent (n=755, 80%). Approximately one-third of patients were active smokers (n=299, 32%) (Table 1).

**Table 1:** Patient Characteristics NSAID: nonsteroidal anti-inflammatory drug

Characteristic	Number of Patients (n) N=940	Healed at 12 Months (n,%) N=815	Nonunion at 12 Months (n,%) N=125
Trial			
SPRINT	626	562 (89.8)	64 (10.2)
FLOW	314	253 (80.6)	61 (19.4)
Age			42.4 (14.5)
Mean (SD)	40.9 (15.6)	40.6 (15.8)	
Gender			
Female	231	203 (87.9)	28 (12.1)
Male	709	612 (86.3)	97 (13.7)
Ethnicity			
Caucasian	755	654 (86.6)	101 (13.4)
African-American	58	46 (79.3)	12 (20.7)
Asian	51	47 (92.2)	4 (7.8)
Hispanic	34	29 (85.3)	5 (14.7)
Native	22	20 (90.9)	2 (9.1)
Other	20	19 (95)	1 (5)
Active Smoker	299	251 (83.9)	48 (16.1)
Non-Smoker	640	563 (88.0)	77 (12.0)
Diabetic	44	38 (86.3)	6 (13.6)
Non-Diabetic	896	777 (86.7)	119 (13.3)
NSAID Use	60	51 (85.0)	9 (15.0)
No NSAID Use	880	764 (86.8)	116 (13.2)

The majority of patients sustained their fractures as a result of a high-energy mechanism (n=667, 71%). Furthermore, approximately half of the patients had open fractures (n=499, 53%) and complex fracture patterns (n=428, 46%) that were either comminuted or segmental. Sixty-seven percent of patients underwent reamed intramedullary nailing (n=632) and few patients had postoperative fracture gaps equal to or exceeding 1 cm (n=44, 5%) (Table 2).

**Table 2:** Injury and Treatment Factors

Characteristic	Number of Patients N=940	Healed at 12 Months (n,%) N=815	Nonunion at 12 Months (n,%) N=125
Mechanism of Injury			
High Energy	667	556 (83.4)	111 (16.6)
Low Energy	273	259 (94.9)	14 (5.1)
Isolated Fracture	509	462 (90.8)	47 (9.2)
Multiple Fractures	431	353 (81.9)	78 (18.1)
Closed Fracture	441	414 (93.9	27 (6.1)
Open Fracture	499	401 (80.4)	98 (19.6)
Type I	104	93	11
Type II	190	151	39
Type IIIA	151	119	32
Type IIIB	54	38	16
Type IIIC	0	0	0
Type of Fracture			
Complex (comminuted/segmental)	428	338 (79.0)	90 (21.0)
Simple	512	477 (93.2)	35 (6.8)
Diaphyseal Location of Fracture			
Proximal	84	66 (78.6)	18 (21.4)
Distal	525	470 (89.5)	55 (10.5)
Middle	325	273 (84.0)	52 (16.0)
Nailing Technique			
Reamed IM Nailing	632	548 (86.7)	84 (13.3)
Unreamed IM Nailing	308	267 (86.7)	41 (13.3)
Postoperative Fracture Gap			
<1cm	896	785 (87.6)	111 (12.4)
≥1cm	44	30 (68.2)	14 (31.8)
Time to surgery from injury in hours, median (IQR)†	12.40 (7.00-24.35)	13.43 (7.30-26.10)	8.72 (6.00-17.65)

PCS scores

The mean PCS score at three months for the entire study cohort was 33.5 (SD 9.0). This overall PCS score was consistent for patients in the SPRINT trial assessed with the SF-36 (33.5, SD 9.1) and patients in the FLOW trial assessed with the SF-12 (33.5, SD 8.9). When assessed by strata, 23% of patients had PCS scores of ≥ 40 (n=219), whereas 73% had scores of 20.0 to 29.9 (n=339) or 30.0 to 39.9 (n=344). Relatively few patients scored less than 20 (n=38) (Table 3).

**Table 3:** PCS Score and Nonunion Risk PCS: physical component summary

SF-36/SF-12 PCS Score	Number of Patients N=940	Healed at 12 Months (n,%) N=815	Nonunion at 12 Months (n,%) N=125
< 20.0	38	29 (76.3)	9 (23.7)
20-29.9	339	285 (84.1)	54 (15.9)
30-39.9	344	300 (87.2)	44 (12.8)
≥40	219	201 (91.8)	18 (8.2)

PCS scores and nonunion

The rate of nonunion in smokers was 16.1% as compared to 12.0% in non-smokers. Patients with open, high energy, and complex fractures had nonunion rates that were approximately three times greater than patients with closed (19.6% vs. 6.1%), low energy (16.6% vs. 5.1%), and simple fractures (21.0% vs. 6.8%), respectively. With regards to surgical factors, reamed and unreamed intramedullary nailing had identical rates of nonunion (13.3%), whereas patients with a postoperative fracture gap of ≥ 1 cm had a nonunion rate of 31.8% as compared to 12.4% in those with a fracture gap of <1 cm. The incidence of nonunion increased with every incremental decrease in PCS score strata. Absolute nonunion risk in patients with PCS scores of ≥ 40 was 8.2%, whereas the risk increased to 12.8% and 15.9% in patients with scores of 30.0-39 and 20.0-29, respectively. Patients with a PCS score of <20 had the greatest risk of nonunion at 23.7%.

When controlling for these risk factors in the multivariable logistic regression analysis, open fractures, complex fracture patterns, and three-month PCS scores were significantly associated with nonunion at 12 months. Open fractures were associated with a greater than 2.5 increase in odds of nonunion as compared to closed injuries (OR 2.77, 95%CI: 1.58-4.83), as were complex fractures compared to simple fractures (OR 2.57, 95%CI: 1.64-4.02). In regards to three-month PCS scores, patients with scores between 20.0 and 29.99 had a nearly two-fold greater risk of nonunion as compared to patients with scores of ≥ 40 or a 6.4% adjusted risk increase (ARI) (ARI: 6.4%, 95% CI: 0.6 - 15.3. OR 1.94, 95%CI: 1.08-3.49), whereas those patients with scores below 20 had an even greater odds of nonunion, with approximately 10% increased absolute risk (ARI: 10.3%, 95% CI: 0.1 - 28.2. OR 2.58, 95%CI: 1.02-6.53) (Table 4).

**Table 4:** Multivariate Logistic Regression for Nonunion at 12 Months (n=940) IM: intramedullary; PCS: physical component summary

Risk Factor	OR (95%CI)	P-value
Open Fracture	2.77 (1.58, 4.83)	<0.001
Complex Fracture	2.57 (1.64, 4.02)	<0.001
Reamed IM Nailing	0.65 (0.40, 1.04)	0.074
Active Smoker	1.39 (0.92, 2.10)	0.113
Fracture Gap ≥1 cm	1.72 (0.85, 3.48)	0.134
3-Month PCS Score		
<20	2.58 (1.02, 6.53)	0.046
20 to <30	1.94 (1.08, 3.49)	0.027
30 to <40	1.52 (0.84, 2.77)	0.167
≥40	1.00	
FLOW TRIAL	1.14 (0.68, 1.91)	0.628

In the sensitivity regression analyses, both interaction terms were nonsignificant, suggesting no difference in the odds of nonunion for reamed versus unreamed nailing irrespective of skin integrity, as well as in the odds of nonunion across PCS strata irrespective of the SF instrument used (SF-36 vs. SF-12).

## Discussion

In this prospective observational study of 940 patients with tibial shaft fractures treated with intramedullary nailing, 13% of patients who had not healed their fractures by three months remained unhealed at one-year postoperatively. Open fractures, complex fracture patterns, and low PCS scores (<30) were significantly associated with nonunion. All three risk factors were associated with a two-fold or greater odds of nonunion.

Although there is previous evidence to corroborate our findings that open and complex fractures are associated with a higher risk of tibia fracture nonunion, we are unaware of any previous studies that have directly evaluated early postoperative function as a prognostic marker for eventual healing status [4,12,20]. Previous evidence evaluating the association between functional outcomes and healing has focused rather on the temporal relationship between functional recovery and fracture healing. Timing to the successful performance of daily activities, such as prolonged walking, running and jumping, has been noted to moderately correlate with timing to fracture healing [23]. Building on such previous work, our current study directly suggests that functional recovery not only has a temporal relationship with fracture healing but that early functional recovery serves as a prognostic indicator for a patient’s ultimate propensity to heal.

Notably, patients in the FLOW trial had nearly double the nonunion rate of patients in the SPRINT trial (19% vs. 10%). This risk difference was most likely attributable to the exclusive enrolment of patients with open fractures in the FLOW trial, as there was no difference in the odds of nonunion between these study cohorts when controlling for open fractures in our regression model (OR 1.14, 95%CI: 0.68,1.91).

Our study has several strengths. First and foremost, this study has a robust sample size of patients stemming from two large, multicenter, randomized controlled trials that were conducted across six countries. Data collection in these trials was done prospectively with quality control checks to ensure accuracy and completeness. Furthermore, both trials had greater than 90% patient follow-up at one year. Second, our chosen measure of physical function is based on a ubiquitous health-related quality of life instrument, with documented validity, reliability, and responsiveness [20,22,25]. The reliability of the SF instruments is of particular importance for our current study, as the utility of a tool for predicting fracture healing is predicated on its widespread reproducibility. Although to our knowledge, there has been no previous precedent for amalgamating PCS scores across the SF-36 and SF-12 instruments, our findings demonstrated consistent findings as expected between the two instruments after sensitivity analysis. Finally, our conclusions regarding the prognostic utility of the SF-36 and SF-12 PCS scores in predicting nonunion are based upon a multivariable regression model in which several known covariates of fracture healing were accounted for.

The primary limitation of our study is attributable to the lack of a gold standard definition of radiographic and clinical fracture healing [26]. In the current study, we relied upon physician judgment at each center to ascertain healing status (bone union or nonunion) at one year based on radiographic findings. Although this is most commonly defined as radiographic healing in three of the four cortices seen on anteroposterior and lateral radiographs, this definition was not put forth as a required diagnostic criterion for the participating trial centers. Some may also question whether the SF-36 and SF-12, which are generic measures of functional status, are sufficiently sensitive for tibial fracture patients. We have compared the Short Musculoskeletal Function Assessment Dysfunction Index (SMFA DI) and the SF-36 PCS scores among 1,319 patients undergoing operative management of tibial fractures. The SMFA DI and SF-36 PCS scores were highly correlated at three, six, and 12 months post-surgical fixation, and the difference in the mean standardized change scores for SMFA DI and SF-36 PCS, from three to 12 months post-surgical fixation, was not statistically significant [27]. Finally, patients with incomplete data were excluded, as data was assumed to be not missing at random, therefore, reducing the certainty in our outcomes.

## Conclusions

In conclusion, a considerable portion of patients with fractures of the tibial shaft treated with intramedullary nailing will fail to heal their fractures at one-year postoperatively. The impact of tibial shaft nonunion on physical and mental health is devastating, such that patients on average would be willing to give up over a third of their remaining lives in exchange for good health. In addition to open injuries and complex fracture patterns, high-risk patients can be identified early in their healing course, in part, by their functional recovery at three months as measured by the PCS scores of the SF-36 and SF-12 instruments. Collectively, the presence of these prognostic markers should lead to increased surveillance and timely management, thereby reducing the potential burden of tibial shaft fracture non-union. 
